# Sexual differences in age-dependent survival and life span of adults in a natural butterfly population

**DOI:** 10.1038/s41598-020-66922-w

**Published:** 2020-06-25

**Authors:** Marcin Sielezniew, Agata Kostro-Ambroziak, Ádám Kőrösi

**Affiliations:** 1 Laboratory of Insect Evolutionary Biology and Ecology, Faculty of Biology, University of Bialystok, Ciołkowskiego 1J, 15-245, Białystok, Poland; 2 MTA-ELTE-MTM Ecology Research Group, Pázmány Péter s. 1/C., Budapest, 1117 Hungary; 3 Theoretical Evolutionary Ecology Group, Department of Animal Ecology and Tropical Biology, Biocenter, University of Würzburg, Emil-Fischer str. 32, 97074 Würzburg, Germany

**Keywords:** Behavioural ecology, Population dynamics, Zoology

## Abstract

Adult survival and longevity in insects are key life-history traits, but their variation between sexes and individuals in natural populations is largely unexplored. Sexual divergence in senescence, the decline in survival with age is also poorly understood. Based on an intensive mark-recapture dataset of the butterfly *Polyommatus daphnis*, we aimed to assess whether adult survival is age-dependent, and to estimate life span distribution and abundance of males and females using Cormack-Jolly-Seber and Jolly-Seber models. Female survival slightly increased with date of emergence and slightly decreased with age, while male survival considerably declined with age. Mean life span of females (12.7 days) was ~50% higher than that of males (8.5 days), but two times higher if only the oldest 5% of each sex was considered (39 vs.19 days). Abundance of females (358 ± 14) and males (359 ± 11) was similar, but peak abundance of males preceded that of females by 11 days. Our results suggest that senescence is much more rapid in males than in females in this butterfly, which is in agreement with sexual selection theory. We also conclude that estimating life span distributions provides much more valuable information on the demography of natural populations than simply reporting the mean life span.

## Introduction

In holometabolous insects, the adult stage is dedicated mostly to reproduction. This stage can be very short in many species which do not feed during this stage at all, but several others are able to acquire some nutrients to increase their fecundity and potential to find a mate, colonize new sites or migrate^1^. Therefore adult survival, and consequently life span, may significantly affect reproductive success, and can be considered as important fitness components^2,3^. There is a growing body of evidence on that senescence, the decline in performance and survival with advancing age^4^, is widespread in wild populations^5^, although most of the studies were conducted on vertebrates. Reproductive senescence means the decline in reproductive output, while actuarial senescence is the decline in survival with age^4^. Theory predicts that sexual selection may produce sex-specific optima for traits that affect survival and ageing rate, often favouring a ‘live fast, die young’ strategy in males, i.e. higher mortality and more rapid ageing than in females^6,7^.

Under natural circumstances, estimating survival and ageing rate is very challenging, particularly for small-bodied, short-living insects^8^, thus insect demography is relatively understudied compared to that of vertebrates^9^. There are only a handful of studies that properly estimated survival and ageing rate of both sexes in natural populations of insects. First Bonduriansky & Brassil^10^ could detect ageing in males of an antler fly. In a wild population of neriid flies, Kawasaki *et al.*^11^ observed higher mortality of males and no sign of ageing in females, while Dukas^12^ provided evidence on senescence in honey bee foragers. Zajitschek *et al.*^13^ also found higher mortality in males of a field cricket population and both sexes showed senescence with a similar ageing rate. Sherratt *et al.*^14^ obtained similar results in a damselfly population, except that ageing rate of males was higher. Most recently, actuarial senescence in field crickets was found to be correlated with phenotypic senescence and affected by population sex-ratio^15,16^. One study that estimated ageing rate in a natural butterfly population detected senescence in both sexes, but did not find significant differences in mortality and ageing rate^17^. The low number of studies and the high variation of their findings suggest a knowledge gap in sex-specific survival and senescence in natural insect populations (for a more detailed review see^18^).

Butterflies are among the most intensively studied insects and they are model organisms in ecological and evolutionary research [e.g.^19^]. Many studies have investigated how environmental factors [e.g.^20-23^], flight activity^24^, adult diet [e.g.^25-27^], or mating history^28,29^ affect the longevity of butterflies. Besides the effects of these factors, such studies have also successfully revealed that adult survival and life span can show large variation both between species^30^, and between or within populations of the same species^31,32^. Most of these studies, however, were conducted under laboratory conditions, and our understanding of life span variation in natural populations is still limited. Conclusions from laboratory studies can be misleading, for example, on the roles of nutrition or sexual selection in shaping senescence through trade-offs (for a thorough review see^18^).

Demographic parameters such as survival and senescence in natural populations can best be studied using the mark-recapture method^8,33^. Butterflies have been the subject of many mark-recapture studies for a long time [see ^34,35^ and references therein], but most of them primarily aimed to estimate population size [e.g.^36^] and/or explored dispersal [for a review see ^37^] or movement within habitat patches [e.g.^38-41^]. Less attention has been paid to survival analysis, and usually only time-dependency of survival was tested, i.e. if survival was constant or whether it changed during the sampling period [e.g.^42^]. Age-dependency of survival (senescence) has been recently demonstrated in natural populations of a butterfly species^17^, and in a lab-reared population under semi-natural conditions^43^. Otherwise many detailed mark-recapture analyses failed to detect age-dependent survival, and only a few studies inferred it, from survivorship curves [e.g.^44^]. Moreover, hardly any study aimed to understand the sexual differences in survival and ageing of butterflies.

The distribution of adult life span in butterfly populations is hardly ever estimated in field studies. Even if survival is properly estimated, most studies only derive an estimation of mean life span in the case of constant survival [see^45^ and references therein]. However, if survival is constant, life span follows a right skewed negative exponential distribution where the mean value does not provide information about the life span of individuals that live much longer than the average, although these individuals may produce considerably more offspring and may be more likely to disperse than short-lived ones^21,22,46^.

We studied a single isolated population of Meleager’s Blue (*Polyommatus daphnis*) butterfly by carrying out an intensive mark-recapture sampling covering the whole flight period. We aimed (i) to assess whether adult survival depended on time since marking (‘age’) or date of marking (‘cohort’); (ii) to unravel sexual differences in survival and ageing rate; (iii) to estimate life span distributions for both sexes and (iv) to estimate daily and total population sizes.

## Materials and Methods

### Study species

The Meleager’s Blue *Polyommatus daphnis* (Denis & Schiffermüller, 1775) is a relatively large lycaenid butterfly (Lepidoptera, Lycaenidae) with a wing span of about 33–37 mm. It shows a striking sexual dimorphism, with males being pale shiny blue, while females are less brightly coloured and occur in two main forms, i.e. a pale sky-blue with broad dark borders, and a dark grey-brown (*f. steeveni)*. Moreover, females are usually somewhat smaller and have a deeply scalloped outer margin of hind wings^47^.

*Polyommatus daphnis* has a Ponto-Mediterranean distribution ranging from NE Spain through Southern and Central Europe to S Ural, Transcaucasus and Iran^47^. The species inhabits nutrient-poor grasslands, usually on calcareous soils, often on hills and mountain slopes up to 2000 m above sea level. Vegetation on the sites is often encroached by scrub and the sites are typically surrounded by woodlands. Across its European range, *P. daphnis* is usually local and not abundant, except on the Balkan Peninsula. This univoltine butterfly is on the wing from mid-June until the end of August, depending on the latitude and altitude of the locality. Caterpillars feed on *Securigera varia* (L.) Lassen and/or *Hippocrepis comosa* L. They are facultatively myrmecophilous, attended by *Lasius, Formica* and *Tapinoma* ants. The butterfly overwinters as an egg or a young larva^47^, but in Poland only the former stage is reported^48^.

### Study site

The study was conducted on a site (N53°02′ E22°55′, ca 120 m a.s.l.) in the Podlasie region (NE Poland), near the Uhowo village close to the Narew National Park. The biotope was relatively heterogeneous and encompassed dry and mesic meadows with diverse herbaceous vegetation and some shrubs. *P. daphnis* was encountered on an area of about 5 ha (i.e. half of the regularly explored area during sampling), but the highest density was within the vicinity of patches of *S. varia* (the only larval food plant in Central Europe) and along dirt roads where the vegetation was generally shorter and rich in nectar plants. The site was bordered by a forest and arable fields, and it had been unmanaged in at least the last five years before the study. The population was located close to the northern limit of the species’ distribution in Poland^48^. We assumed that it was an isolated population since we did not find any other occurrence of the butterfly in the vicinity (i.e. in the radius of about 3 km) and no observations were reported from the surrounding area.

### Data collection

The population was sampled using mark-release-recapture (MRR) on 38 days between 3 July and 18 August 2014. We aimed to cover the entire flight period of *P. daphnis*, and started sampling when only a few males were on wing yet. The site was visited almost every day, weather permitting, except at the end of the flight period when the frequency of visits was lower. The weather conditions were generally favourable and stable. The vast majority of days during the flight period were sunny with temperatures 22–32 °C and only in the beginning there were three rainy days (July 5, 11, and 12) that did not allow sampling. Each time, 1–3 people were engaged in sampling, and spent five hours per day on the site on average. Butterflies were captured by an entomological net, marked on the underside of their hind-wings with unique numbers (Fig. S1d) using a fine-tipped pen with waterproof, non-toxic ink, and released at the place of capture. Date, time and GPS coordinates of each (re)capture, as well as the sex of the individuals were recorded. Sampling was finished when only a single worn individual was captured during a two-hour sampling session on 18 August. Moreover, on the following day weather conditions became unfavourable for a longer period. Additionally we mapped all patche*s* of *S. varia* at the site using GPS.

### Data analysis

Mark-recapture data were analysed using Cormack–Jolly–Seber (CJS) and Jolly–Seber (JS) models. The CJS model has two parameters, apparent survival rate (*φ*) and recapture probability (*p*). Apparent survival (*φ*) is the probability that a marked individual that is alive at occasion *i* will be alive and present in the population at occasion *i* + 1, hence this parameter applies to sampling intervals. (In single-site MRR studies, we cannot distinguish between emigration and death of individuals, therefore we estimate apparent survival, i.e. the probability that an individual survived and did not emigrate. However, in isolated populations such as our study population, apparent survival is reasonably a very good proxy of true survival.) Recapture probability means the probability that a marked individual that is present alive in the population at occasion *i* will be captured, and it applies to sampling occasions. In the formulation that we used, known as POPAN, the JS model includes two additional parameters: size of a ‘superpopulation’ (*N*), i.e. the number of all individuals that were in the population (and available for capture) during any of the sampling occasions, and probability of entry (*pent*), which represents the probability that individuals of this hypothetical ‘superpopulation’ enter the population and become available for capture between occasion *i* and *i* + 1^49^. The JS model assumes that survival and capture probabilities are equal for both marked and unmarked individuals of a population, thus it enables estimation of the population size^50^. We analysed the data on males and females separately, and in the case of females, we also involved wing colour type (blue vs. brown) as a grouping factor.

We tested the dependency of model parameters from covariates such as time, age and cohort, by constructing a set of 35 CJS models: (i) a parameter could be constant, i.e. it had the same value for all sampling occasions/intervals (‘~1’); (ii) a parameter could change with time, i.e. it could have different values at each sampling occasion/interval (‘~time’); (iii) a parameter could change during the sampling period linearly (‘~Time_lin’); (iv) a parameter could linearly change with cohorts (a cohort is the group of individuals marked on the same sampling occasion) (‘~Cohort’); and (v) a parameter could change with the time elapsed after marking (‘~Age’) (see also^17^). We can consider the latter one as an ‘Age’-model if we assume that all individuals were marked soon after eclosion. Given our high sampling intensity this assumption is likely not violated. We built three ‘Age’-models for apparent survival. In the logistic model, the logit-transformed survival changed linearly with age (time since marking), in the Gompertz-model the loglog-transformed survival changed with age, while in the Weibull-model the loglog-transformed survival changed with ln(age) linearly^51^. For recapture probability, we used the logistic model only. In ‘~Time_lin’ and ‘~Cohort’ models (iii & iv), the logit-transformed parameters (survival *φ* and recapture probability *p*) were related linearly with time and cohort number, respectively. In all these models (iii, iv & v) an intercept and a slope are estimated for each demographic parameter.

Since marked and unmarked animals are not distinguished in JS models, here ‘Age’ and ‘Cohort’ models are not applicable. In JS models, three parameters can be constrained (*φ*, *p* and *pent*; *N* is constant), so we fitted twenty-seven models to each dataset. We performed a model selection based on AICc values^52,53^. We carried out ‘Goodnes of Fit’ (GOF) tests on the CJS models using different approaches (‘RELEASE’ and bootstrap). We estimated the overdispersion parameter *ĉ* and adjusted the model estimates with it when it was necessary (see the description of methods and results of GOF-tests in Supplementary information). Model construction was conducted using the ‘RMark’ package version 2.2.7^54^ within the R statistical software 3.6.3^55^, which provides a flexible R-like interface to the core routines of MARK 9.0 software^49^ that performs model fitting and estimations. GOF-tests were performed in MARK 9.0.

Based on the estimates of apparent survival rate (*φ*) of the CJS models, we calculated mean life span and plotted life span distribution for males and females. In the case of a constant survival rate, life span follows an exponential distribution, and mean life span can be estimated as (1−*φ*)^−1^−0.5^56^. In the case of age-dependent survival, we calculated the probability of death at a certain age for each round number of days between 0 and 50, and used this distribution to calculate mean life span. In the case of cohort-dependent survival, we calculated an average survival weighted by the number of individuals belonging to each cohort. In all calculations, the day of marking was the zero day of life.

## Results

Although the sampling period consisted of 38 occasions, no females were captured on the first occasion, and no males were captured on the last five occasions after 10 August. Thus encounter histories consisted of 37 and 33 occasions for females and males, respectively, and the curves of cumulative numbers of marked butterflies also suggest that sampling covered the whole flight period (Fig. S2). Altogether 258 females (44 blue-coloured) and 280 males were marked, 67% of female and 75% of male butterflies were recaptured at least once: 173 females were recaptured on 486 occasions and 210 males on 677 occasions (counting only recaptures on different days). Females were recaptured 1.9 times on average (SD = 2.3), while males were recaptured 2.4 times on average (SD = 2.5). One of the females was captured on 16 different days, and one male on 15 (Fig. S3), and the longest period between first and last captures of the same individual was 38 days for females and 30 days for males. In the first ca. three weeks of the sampling period, the number of captured and marked males was higher, but in the second half females outnumbered the males (Fig. S4).

Butterflies were detected most often (in ca. 50% of capture events) on flowers during nectaring. They visited a total of 25 different plant species, but 85% of the visits were observed on five species and *Lotus corniculatus* was the most frequently visited by both sexes. Males were more often observed flying than females (38% vs. 25%, respectively), while females rested/basked more often. Other behaviours were directly related to reproduction (4.9% and 12.6% of males and females, respectively), i.e. courtship (2.8%) and mating (2.1%) for males, and courtship (3.4%) mating (3.0%) and oviposition (6.2%) for females. We observed 23 matings, 20 of them in the first two weeks of the sampling period. Sex specific differences in behaviour were highly significant both when four main activities were distinguished (‘nectaring’, ‘flight’, ‘resting/basking’ and ‘reproduction’) (*p* ≪ 0.0001, *χ*^2^ = 74.6) and when only ‘flight and ‘other behaviours’ were taken into consideration (*p* ≪ 0.0001, *χ*^2^ = 35.9). Interestingly, no predation on *P. daphnis* was observed during our field work.

### CJS models and life span estimation

**Figure 1:**
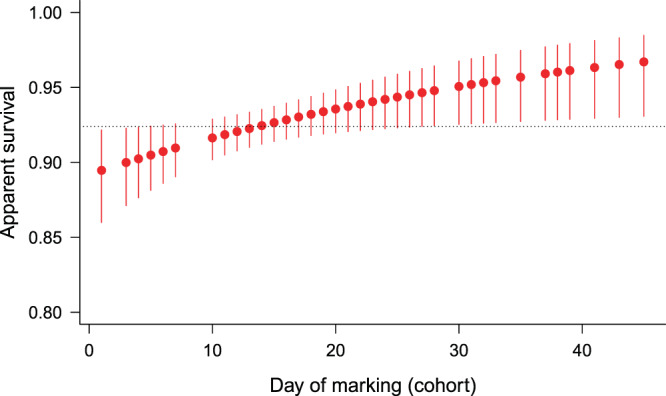
Estimated apparent survival rate for females in relation to days of marking (cohort) from the most supported model *φ*(~Cohort)*p*(~Cohort). Error bars represent 95% confidence intervals. Dotted line shows the weighted average.

**Figure 2:**
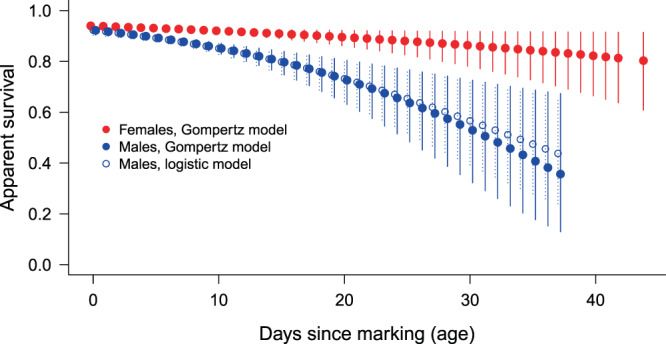
Estimated apparent survival rate for males (*φ*(~Age)*p*(~time) model) and females (*φ*(~Age)*p*(~Cohort) model) in relation to the number of days since marking (age). For males, estimates of both the Gompertz and the logistic models are shown. Error bars represent 95% confidence intervals.

**Figure 3:**
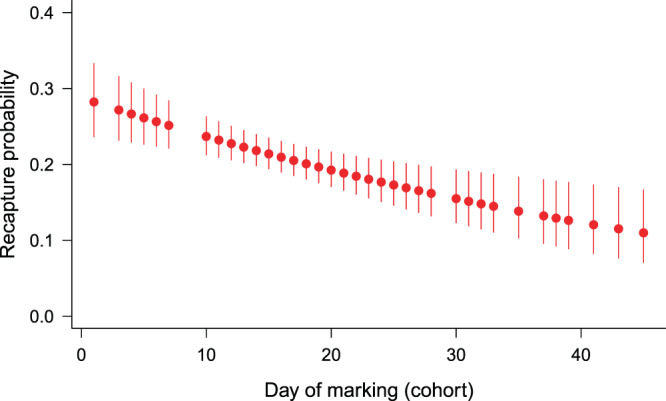
Estimated recapture probabilities of females in relation to days of marking (cohort) from the most supported model *φ*(~Cohort)*p*(~Cohort). Error bars represent 95% confidence intervals.

We did not find any significant differences in CJS model parameters between blue and brown-coloured females, so we pooled all female data. We adjusted the models with *ĉ*=1.07 estimated by the GOF-tests (Supplementary Information, Table S1). The model selection procedure did not support one single model as best, but there were four models with ΔQAICc < 2. In Table 1, we show the five most supported models. In the first two models, apparent survival rate (*φ*) increased with cohort number (females marked earlier had lower survival), in the third and fourth models (Gompertz and logistic) survival declined with time since marking (age), but the difference between the two models was negligible. Likelihood-ratio tests suggested that models with cohort-dependent survival explained the data significantly better than models with constant survival, but for the age effect the evidence was weaker (Table 2). The change in survival with cohort and age was quite low (Table 1, Fig. 1 & Fig. 2). Recapture probability decreased linearly with cohort number or time in all the five models and the estimates of different models were very similar (Table 1, Fig. 3).

**Table 1:** The most-supported CJS models and their estimates of apparent survival and recapture probability for each sex. In models where any of the parameters changed linearly with age, cohort or time, the range of estimates is shown. In the case of single estimates, the SE is shown in brackets. In case of females QAICc values were calculated due to *ĉ* adjustment.

Sex	Model specification	Δ(Q)AICc	(Q)AICc weight	Estimates of survival φ	Weighted average of estimated survival φ	Estimates of recapture probability p
Female	Phi(~Cohort)p(~Cohort)	0.00	0.195	0.895–0.967	0.924	0.282–0.110
	Phi(~Cohort)p(~Time_lin)	0.02	0.193	0.901–0.968	0.928	0.297–0.137
	Phi(~Age_Gompertz)p(~Cohort)	0.84	0.128	0.940–0.803	—	0.268–0.126
	Phi(~Age_logistic)p(~Cohort)	0.91	0.124	0.940–0.809	—	0.268–0.126
	Phi(~1)p(~Time_lin)	2.90	0.050	0.927 (0.006)	—	0.283–0.152
Male	Phi(~Age_Gompertz)p(~time)	0.00	0.511	0.922–0.357	—	see Fig. 4
	Phi(~Age_logistic)p(~time)	0.16	0.472	0.923–0.438	—

**Table 2:** Likelihood-ratio tests between models with cohort- or age-dependent survival vs. constant survival in females. The number in brackets after model name is the model rank after model selection. *P*-values < 0.05 are in bold.

Reduced model	General model	χ2	df	p-value
φ(~1)p(~Cohort) (8)	φ(~Cohort)p(~Cohort) (1)	5.477	1	**0.0193**
φ(~1)p(~Time_lin) (5)	φ(~Cohort)p(~Time_lin) (2)	4.907	1	**0.0268**
φ(~1)p(~Cohort) (8)	φ(~Age_Gompertz)p(~Cohort) (3)	4.635	1	**0.0313**
φ(~1)p(~Time_lin) (5)	φ(~Age_Gompertz)p(~Time_lin) (6)	1.675	1	0.1955

**Figure 4:**
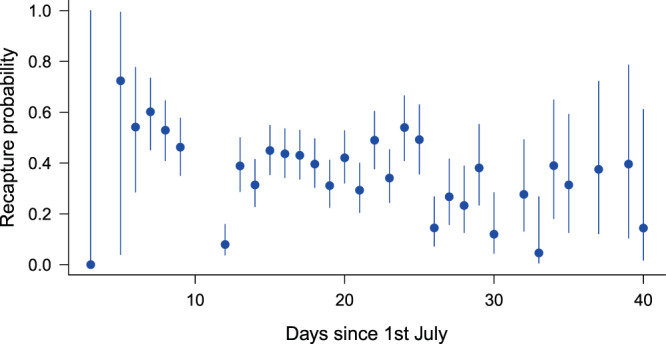
Estimated recapture probabilities of males in relation to time from the most supported model *φ*(~AgeGompertz)*p*(~time). Error bars represent 95% confidence intervals.

In the case of males, the GOF-tests suggested no overdispersion (*ĉ* ≈ 1) and we found that the Gompertz and the logistic ‘Age’-models highly outperformed all others. In these most-supported models, apparent survival rate (*φ*) decreased with age, while recapture probability changed with time non-linearly (Table 1, Fig. 2 & Fig 4). Survival declined more rapidly with age in the Gompertz-model, but this manifested at older ages (>20 days) only (Fig. 2). Male survival declined much more rapidly with age than female survival (Fig. 2) and initial survival (at age = 0) was also lower in males.

**Figure 5:**
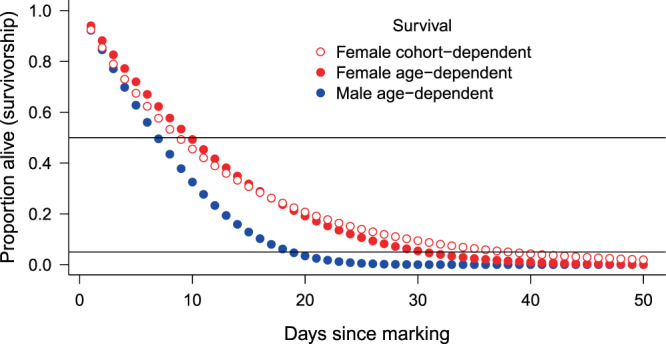
Proportion of butterflies alive in relation to age. Horizontal lines at *y* = 0.5 and *y* = 0.05 represent the median and 95% percentile age of survivors.

**Figure 6:**
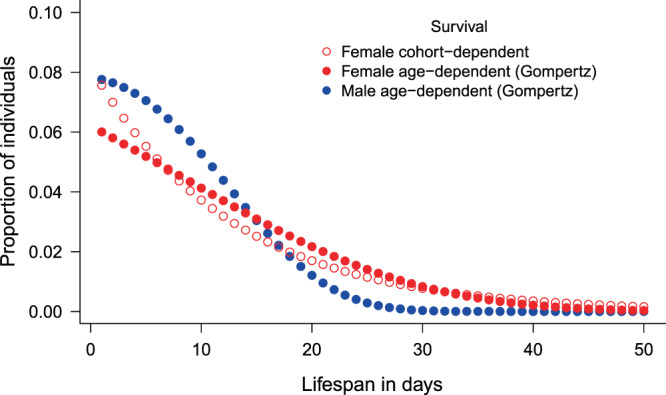
Probability distribution of life span of both sexes.

The weighted average of survival estimates of cohort-dependent models for females provided very similar results to the constant survival model (Table 1). Therefore, we used two scenarios for the life span distribution calculations for females: (i) constant survival rate (*φ* = 0.924, the weighted average from the most supported model *φ*(~Cohort)*p*(~Cohort)) and (ii) age-dependent survival (according to the *φ*(~AgeGompertz)*p*(~Cohort) model). We found considerable differences in the survivorship curves (cumulative proportion of survivors) between sexes, and much less between the two scenarios for females (Fig. 5). Median life span did not show a big difference (7 days for males, 10 and 9 days for females with age- and cohort-dependent survival, respectively). Mean life span was calculated as 8.5 days for males, and 12.4 and 12.7 days for females with age- and cohort- dependent survival, respectively. The survivorship curves suggest that ~95% of males died by the age of 19 days, and hardly any male survived longer than ~25 days. In contrast, five percent of the females survived for at least 30 days in the case of age-dependent, and for at least 38 days in the case of cohort-dependent survival (Fig. 5). We calculated mean life span for the oldest roughly 5% of males as 19.3 days, whereas it was 31.9 and 39.3 days for females with age-dependent survival and cohort-dependent survival, respectively. Life span distributions also showed large differences between sexes: short life spans are more frequent and long life spans more rare in males than in females (Fig. 6).

### JS models and population size estimation

**Figure 7:**
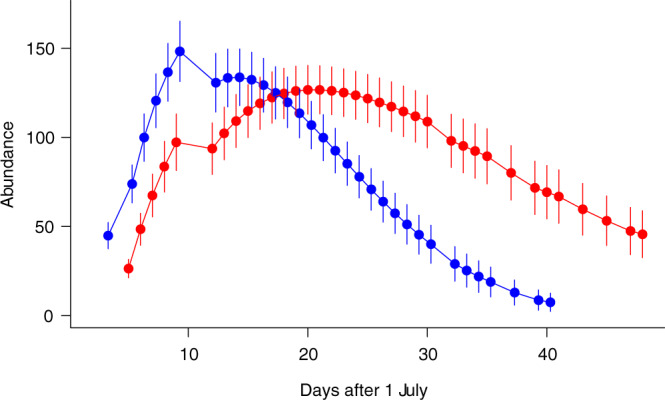
Estimates of daily population size for females (red circles) and males (blue circles). Error bars represent 95% CIs.

In the most supported JS model for females, apparent survival rate was constant (0.926) and capture probability changed with time non-linearly, while probability of entry decreased linearly with time. The size of the ‘superpopulation’ (±SE) was 341.2 (±12.63), the gross population estimate was 357.6 (13.8). For males, apparent survival rate decreased linearly and capture probability changed non-linearly, while probability of entry decreased linearly with time. The size of the ‘superpopulation’ (±SE) was 338.6 (±9.88), the gross population was estimated as 358.6 (11.3). Estimations of daily population size suggest that peak abundance of males (10 July) preceded that of females (21 July) by 11 days, and that female abundance was still above zero at the end of the sampling period (Fig. 7).

## Discussion

Variation in survival and senescence between sexes in natural populations of insects is a poorly studied area of ecological and evolutionary research. In the present study, we found strong evidence on senescence in male butterflies, while alternative models better explained survival in females. Moreover, ageing rate was higher in males, although their initial survival was only slightly lower than in females. Consequently, estimated life span distributions of male and female butterflies were markedly different as females had longer life spans. Our findings are mostly in agreement with the handful of field studies on sexual differences in survival and senescence in insects. Although initial survival of males was not much lower than for females, the much more rapid ageing of males resulted in shorter life spans [see^10,11,13,14^^,^^17^^,^^19-21^].

The sexual difference in ageing rate and life span distributions can be interpreted in the light of sexual selection^57,58^. Because of the divergence in sex roles stemming from anisogamy, males are expected to pursue a high-risk reproductive strategy with the potential for a high yield over short time periods, while females are expected to follow low-risk strategies with moderate yield over extended time periods^6^. It is generally predicted, that males can benefit by sacrificing longevity for the possibility of enhanced mating success, whereas females cannot gain as much because their fitness is limited by the time investment in offspring production^7^. Higher mortality and ageing rate of males can, for example, be a result of combat that is often involved in male reproductive strategies and causes somatic damage. Reduced life expectancy may also drive the evolution of more rapid ageing in males^59–61^. Furthermore, males may also allocate more resources to secondary sexual traits thus lowering viability for enhanced sexual performance.

In butterflies, the reproductive success of males depends on the number of females they can mate with^62,63^. The susceptibility of females for mating can decline after their first mating, especially if they are monandrous^64^. For example, in the monandrous butterfly *Pararge aegeria*, males’ longevity was significantly shorter in populations from Sweden where males and females emerge synchronously, while no difference was found in a population from Madeira where butterflies emerge continuously through the year^31^. Males are often selected to emerge before females (‘protandry’) and usually compete for females, which can manifest even in contest fights^65^. These combat fights may cause unrepairable damage of the exoskeleton, resulting in higher mortality of males. In our study species, we did not observe any contest fight, but the frequency of observed matings clearly declined during the season.

Mating system can affect the sexual divergence in survival and longevity. Wiklund *et al.*^66^ found higher mortality and shorter longevity in males than in females in a monandrous butterfly species, while male and female life spans were similar in a polyandrous species. During sampling, we observed a relatively high number of mating pairs, but we did not record any individual mating more than once, and all females spotted in copula looked very freshly emerged. Hence monandry is the more likely mating system for *P. daphnis*, especially since this is also a typical system for species with a clear protandry^67^. In the presently studied population, males emerged and reached peak abundance distinctly earlier than females. Protandry is frequently observed in butterfly populations [e.g.^17^^,^^42,68,69^] and is thought to be an adaptive strategy to maximize the reproductive success of both males and females^70,71^.

During the reproductive season, after the peak abundance of females, the chance for a male to find susceptible females for mating may decline rapidly^62^. Therefore, we suggest that males are strongly selected to allocate as many resources as possible into the first few days of their life when competition for virgin (susceptible) females is the strongest. On the other hand, females do not compete for mates and they are likely to be time-limited in their egg-laying, as it was demonstrated in some butterfly species^72,73^. Thus, fecundity and the reproductive success of females are expected to be positively related to life span. In other words, if host plants for egg-laying are available for longer than the flight period (as in our case) then phenologies of the main resources for females and males are very different, and females are selected for a longer life span. Moreover, mate searching and contest competition among males may energetically be so costly^62,74^ that this causes a more rapid decline in their survival with age. In our study, higher recapture probability of males may stem from that they are often engaged in patrol flights searching for females. We hypothesize, that males’ elevated flight activity can cause higher predation risk (mostly by spiders) and faster somatic deterioration than in females, possibly driving more rapid ageing in males. In order to find mates, males can also be more willing to fly at suboptimal temperatures that may load them with considerable physiological costs^75,76^.

The resource allocation framework can also help us to understand sexual differences in senescence^77^. Due to their different selective pressures, males and females may allocate the resources accumulated during the larval phase to different life-history traits (e.g. flight muscles and flight vs. oocytes) and energy reserves may be depleted at different rates^78^. Moreover, males may transfer nutrients as a parental investment to females during copulation, which can further deteriorate male survival^79^. To unravel these sex-specific patterns in resource allocation, however, further research is inevitable.

Phenological discrepancy between sexes can be compensated for by longevity of males, which is positively correlated with the degree of protandry^67^. Although in our study the estimated life span of males was lower than for females, it was still relatively high compared to other lycaenids [cf^17,80,81^]. Furthermore, we did not find any differences in parameters between the two colour forms of females in contrast to Turlure *et al.*^82^, who studied polymorphism in *B. eunomia* and found that less-numerous andromorph females had significantly lower catchability and lower daily survival than gynomorph females. In the damselfly *Coenagrion puella*, Sherratt *et al.*^14^ detected no difference in the survival of two colour forms of females. Butterfly wing patterns are influenced by a few different adaptation pressures^83^, and further studies of *P. daphnis* could contribute to a better understanding of this issue.

Hitherto, mark-recapture studies on butterfly populations have mostly aimed at estimation of population size [e.g.^36,56^] and dispersal parameters [for a review see^37^]. Much less attention has been paid to survival estimations during data analysis, most studies strove to exclude age-dependency. Our study clearly demonstrates that this approach leads to a simplistic view of life span distribution, and hampers the detection of important intra- and inter-specific differences. Recently, Bubová *et al.*^45^ reviewed the literature on life span estimations of European butterflies, and related mean life span to the length of the flight period for each species. It was revealed that species with shorter mean life spans compared to flight period lengths are more likely to be of concern as regards conservation. However, only estimates of mean life span were used in this meta-analysis, and no more than ca. 10% difference in life span estimates between males and females was found for the total number of 50 butterfly species reviewed. Our results highlight that large inter-sexual differences may occur in life span distributions that may affect the temporal fragmentation of the population^45^, and operational sex ratio, since males spend a much shorter time in the population than females. Furthermore, the life span of ca. 5% of females was almost as long as the flight period, suggesting that the estimation of temporal fragmentation should be more sophisticated than the simple calculation of ratio of flight period length and mean life span.

We note that in single site mark-recapture studies, emigration probability and mortality cannot be estimated independently. However, our study population was highly isolated, no other populations were known in the vicinity, and we did not observe butterflies leaving the sampling area. Thus we assume that emigration probability, which may affect estimated adult residency time considerably [e.g.^84^], was so low that the apparent survival estimated by our models was very close to true survival [see also^17^]. We also note that the longest timespan between first and last captures of the same individual was 38 days in females and 30 days in males, both of which highly exceeded the average estimated life span. Indeed, the observed time span between first and last captures was higher for some individuals in our studied population, than the 95 percentile of the life span calculated from survival estimates. Hence we suggest that those studies in which this timespan was regarded as a mean life span [e.g.^85,86^] should be treated cautiously. Instead, we recommend proper modeling of survival and estimation of life span distributions, such as we performed in our present study.

## Supplementary information

Supplementary Information.

## References

[1] Carey JR (2001). Insect biodemography. Annu. Rev. Entomol..

[2] Bronikowski AM, Promislow DEL (2005). Testing evolutionary theories of aging in wild populations. Trends Ecol. Evol..

[3] Stearns, S. C. The evolution of life histories. (Oxford University Press, Oxford, 1992).

[4] Monaghan P, Charmantier A, Nussey DH, Ricklefs RE (2008). The evolutionary ecology of senescence. Funct. Ecol..

[5] Nussey DH, Froy H, Lemaitre J-F, Gaillard J-M, Austad SN (2013). Senescence in natural populations of animals: Widespread evidence and its implications for bio-gerontology. Ageing Res. Rev..

[6] Vinogradov AE (1998). Male reproductive strategy and decreased longevity. Acta Biotheor..

[7] Bonduriansky R, Maklakov A, Zajitschek F, Brooks R (2008). Sexual selection, sexual conflict and the evolution of ageing and life span. Funct. Ecol..

[8] Nussey DH, Coulson T, Festa-Bianchet M, Gaillard J-M (2008). Measuring senescence in wild animal populations: towards a longitudinal approach. Funct. Ecol..

[9] Salguero-Gomez R (2016). COMADRE: a global data base of animal demography. J. Anim. Ecol..

[10] Bonduriansky R, Brassil CE (2002). Rapid and costly ageing in wild male flies. Nature.

[11] Kawasaki N, Brassil C, Brooks R, Bonduriansky R (2008). Environmental effects on the expression of life span and aging: an extreme contrast between wild and captive cohorts of *Telostylinus angusticollis* (Diptera: Neriidae). Am. Nat..

[12] Dukas R (2008). Mortality rates of honey bees in the wild. Insect. Soc..

[13] Zajitschek F, Brassil CE, Bonduriansky R, Brooks R (2009). Sex effects on life span and senescence in the wild when dates of birth and death are unknown. Ecology.

[14] Sherratt TN (2010). Empirical evidence of senescence in adult damselflies (Odonata: Zygoptera). J. Anim. Ecol..

[15] Rodríguez-Muñoz R (2019). Comparing individual and population measures of senescence across 10 years in a wild insect population. Evolution.

[16] Rodríguez-Muñoz R (2019). Slower senescence in a wild insect population in years with a more female-biased sex ratio. Proc. R. Soc. B.

[17] Osváth-Ferencz (2017). Population demography of the endangered large blue butterfly *Maculinea arion* in Europe. J. Insect Conserv..

[18] Zajitschek F, Zajitschek S, Bonduriansky R (2020). Senescence in wild insects: Key questions and challenges. Funct. Ecol..

[19] Boggs, C. L., Watt, W. B. & Ehrlich, P. R. Butterflies: Ecology and Evolution Taking Flight. (University of Chicago Press, Chicago, 2003).

[20] Haeler E, Fiedler K, Grill A (2014). What prolongs a butterfly’s life?: trade-offs between dormancy, fecundity and body size. PloS One.

[21] Karl I, Fischer K (2009). Altitudinal and environmental variation in lifespan in the Copper butterfly Lycaena tityrus. Funct. Ecol..

[22] Karlsson B, Wiklund C (2005). Butterfly life history and temperature adaptations; dry open habitats select for increased fecundity and longevity. J. Anim. Ecol..

[23] Sielezniew (2019). Habitat-related differences in the adult longevity of two ecotypes of a specialized butterfly. J. Zool..

[24] Gibbs M, Van Dyck H (2010). Butterfly flight activity affects reproductive performance and longevity relative to landscape structure. Oecologia.

[25] Cahenzli F, Erhardt A (2012). Nectar sugars enhance fitness in male *Coenonympha pamphilus* butterflies by increasing longevity or realized reproduction. Oikos.

[26] Molleman F (2008). Adult diet affects lifespan and reproduction of the fruit-feeding butterfly *Charaxes fulvescens*. Entomol. Exp. Appl..

[27] Molleman F, Ding J, Boggs C, Carey JR, Arlet ME (2009). Does dietary restriction reduce life span in male fruit-feeding butterflies?. Exp. Gerontol..

[28] Cordero C (2000). Trade-off between fitness components in males of the polygynous butterfly Callophrys xami (Lycaenidae): the effect of multiple mating on longevity. Behav. Ecol. Sociobiol..

[29] Kawagoe T, Suzuki N, Matsumoto K (2001). Multiple mating reduces longevity of females of the windmill butterfly *Atrophaneura alcinous*. Ecol. Entomol..

[30] Beck J, Fiedler K (2009). Adult life spans of butterflies (Lepidoptera: Papilionoidea + Hesperioidea): broadscale contingencies with adult and larval traits in multi-species comparisons. Biol. J. Linn. Soc..

[31] Gotthard K, Nylin S, Wiklund C (2000). Mating opportunity and the evolution of sex-specific mortality rates in a butterfly. Oecologia.

[32] Bauerfeind SS, Perlick JEC, Fischer K (2009). Disentangling environmental effects on adult life span in a butterfly across the metamorphic boundary. Exp. Gerontol..

[33] McCrea, R. S. & Morgan, B. J. T. Analysis of Capture-Recapture Data. (CRC Press, Taylor & Francis Group, Boca Raton, 2014).

[34] Ehrlich, P. R. & Hanski, I. On the Wings of Checkerspots: A Model System for Population Biology. (Oxford University Press, USA, 2004).

[35] Settele, J., Shreeve, T., Konvička, M. & Van Dyck, H. Ecology of Butterflies in Europe. (Cambridge University Press, Cambridge, 2009).

[36] Turlure C, Pe’er G, Baguette M, Schtickzelle N (2018). A simplified mark-release-recapture protocol to improve the cost-effectiveness of repeated population size quantification. Methods Ecol. Evol..

[37] Stevens VM, Turlure C, Baguette M (2010). A meta-analysis of dispersal in butterflies. Biol. Rev..

[38] Auckland JN, Debinski DM, Clark WR (2004). Survival, movement, and resource-use of the butterfly *Parnassius clodius*. Ecol. Entomol..

[39] Kőrösi Á, Örvössy N, Batáry P, Harnos A, Peregovits L (2012). Different habitat selection by two sympatric *Maculinea* butterflies at small spatial scale. Insect Conserv. Diver.

[40] Nowicki P (2019). What keeps “living dead” alive: demography of a small and isolated population of *Maculinea* (*=Phengaris*) *alcon*. J. Insect Conserv..

[41] Skórka P (2013). Different flight behaviour of the endangered scarce large blue butterfly *Phengaris teleius* (Lepidoptera: Lycaenidae) within and outside its habitat patches. Landsc. Ecol..

[42] Schtickzelle N, Le Boulengé E, Baguette M (2002). Metapopulation dynamics of the bog fritillary butterfly: demographic processes in a patchy population. Oikos.

[43] Zheng C, Ovaskainen O, Saastamoinen M, Hanski I (2007). Age‐dependent survival analyzed with Bayesian models of mark–recapture data. Ecology.

[44] Brakefield PM (1982). Ecological studies on the butterfly *Maniola jurtina* in Britain. II. Population dynamics: the present position. J. Anim. Ecol..

[45] Bubová T, Kulma M, Vrabec V, Nowicki P (2016). Adult longevity and its relationship with conservation status in European butterflies. J. Insect Conserv..

[46] Kahuthia-Gathu R, Löhr B, Poehling HM (2008). Development and reproductive potential of diamondback moth *Plutella xylostella* (Lepidoptera: Plutellidae) on cultivated and wild crucifer species in Kenya. Int. J. Trop. Insect Sci..

[47] Tolman, T. & Lewington, R. Collins Butterfly Guide. (Harper Collins Publishers, London, 2009).

[48] Buszko, J. & Masłowski, J. Motyle dzienne Polski. (Wydawnictwo „Koliber”, Nowy Sącz, 2015).

[49] White GC, Burnham KP (1999). Program MARK: Survival estimation from populations of marked animals. Bird Study.

[50] Schwarz CJ, Arnason AN (1996). A general methodology for the analysis of capture-recapture experiments in open populations. Biometrics.

[51] Gaillard J-M, Viallefont A, Loison A, Festa-Bianchet M (2004). Assessing senescence patterns in populations of large mammals. Anim. Biodiv. Conserv..

[52] Burnham, K. P. & Anderson, D. R. Model selection and multimodel inference: a practical information-theoretic approach. Second Edition. (Springer-Verlag, Berlin, 2002).

[53] Hurvich CM, Tsai C (1989). Regression and time series model selection in small samples. Biometrika.

[54] Laake, J. L. RMark: an R interface for analysis of capture-recapture data with MARK. Alaska Fisheries Science Center, NOAA, National Marine Fisheries Service, Seattle: AFSC Processed Report 2013-01 (2013).

[55] R Core Team. R: A language and environment for statistical computing. R Foundation for Statistical Computing, Vienna, Austria. https://www.R-project.org/ (2016).

[56] Nowicki P (2005). Less input same output: simplified approach for population size assessment in Lepidoptera. Popul. Ecol..

[57] Parker, G. A. Sexual selection and sexual conflict. In *Sexual selection and reproductive competition in insects* (ed. Blum, M. S. & Blum, N. A.) 123–166 (Academic Press, New York, 1979).

[58] Parker GA (2006). Sexual conflict over mating and fertilization: an overview. Philos. Trans. R. Soc. B.

[59] Williams GC (1957). Pleiotropy, natural selection, and the evolution of senescence. Evolution.

[60] Hamilton WD (1966). The moulding of senescence by natural selection. J. Theor. Biol..

[61] Kirkwood TBL, Rose MR (1991). Evolution of senescence: late survival sacrificed for reproduction. Philos. Trans. R. Soc. B.

[62] Wickman, P.-O. Mating behaviour in butterflies. In Ecology of Butterflies in Europe (ed. Settele, J., Shreeve, T., Konvička, M. & Van Dyck, H.) 17–28 (Cambridge University Press, Cambridge, 2009).

[63] Wiklund, C. Sexual selection and the evolution of butterfly mating systems. In Butterflies: Ecology and Evolution Taking Flight (ed. Boggs, C. L., Watt, W. B. & Ehrlich, P. R.) 67–90 (University of Chicago Press, Chicago, 2003).

[64] Rutowski, R. L. Sexual dimorphism, mating systems and ecology in butterflies. In The evolution of mating systems in insects and arachnids (ed. Choe, J. C. & Crespi, B. J.) 257–272 (Cambridge University Press, Cambridge, 1997).

[65] Kemp DJ, Wiklund C (2001). Fighting without weaponry: a review of male-male contest competition in butterflies. Behav. Ecol. Sociobiol..

[66] Wiklund, C., Gotthard, K. & Nylin, S. Mating system and the evolution of sex-specific mortality rates in two nymphalid butterflies. *Proc. R. Soc. B* **270**, 1823–1828 (2003). 10.1098/rspb.2003.2437 PMC1691446 12964985

[67] Wiklund C, Fagerström T (1977). Why do males emerge before females?. Oecologia.

[68] Örvössy N, Kőrösi Á, Batáry P, Vozár Á, Peregovits L (2013). Potential metapopulation structure and the effects of habitat quality on population size of the endangered False Ringlet butterfly. J. Insect Conserv..

[69] Zimmermann K, Fric Z, Filipová L, Konvička M (2005). Adult demography, dispersal and behaviour of Brenthis ino (Lepidoptera: Nymphalidae): how to be a successful wetland butterfly. Eur. J. Entomol..

[70] Fagerström T, Wiklund C (1982). Why do males emerge before females? Protandry as a mating strategy in male and female butterflies. Oecologia.

[71] Zonneveld C (1992). Polyandry and protandry in butterflies. Bull. Math. Biol..

[72] Doak P, Kareiva P, Kingsolver J (2006). Fitness consequences of choosy oviposition for a time-limited butterfly. Ecology.

[73] Kőrösi Á, Örvössy N, Batáry P, Kövér S, Peregovits L (2008). Restricted within-habitat movement and time-constrained egg laying of female *Maculinea rebeli* butterflies. Oecologia.

[74] Kemp DJ, Wiklund C, Van Dyck H (2006). Contest behaviour in the speckled wood butterfly (*Pararge aegeria*): Seasonal phenotypic plasticity and the functional significance of flight performance. Behav. Ecol. Sociobiol..

[75] Berwaerts K, Van Dyck H (2004). Take-off performance under optimal and suboptimal thermal conditions in the butterfly *Pararge aegeria*. Oecologia.

[76] Merckx T, Karlsson B, Van Dyck H (2006). Sex- and landscape-related differences in flight ability under suboptimal temperatures in a woodland butterfly. Funct. Ecol..

[77] Boggs CL (2009). Understanding insect life histories and senescence through a resource allocation lens. Funct. Ecol..

[78] Vande Velde L, Van Dyck H (2013). Lipid economy, flight activity and reproductive behaviour in the speckled wood butterfly: on the energetic cost of territory holding. Oikos.

[79] Bergström J, Wiklund C (2002). Effects of size and nuptial gifts on butterfly reproduction: can females compensate for a smaller size through male-derived nutrients?. Behav. Ecol. Sociobiol..

[80] Nowicki P, Witek M, Skórka P, Settele J, Woyciechowski M (2005). Population ecology of the endangered butterflies Maculinea teleius and M. nausithous, and its implications for conservation. Popul. Ecol..

[81] Timuș N, Craioveanu C, Sitaru C, Rus A, Rákosy L (2013). Differences in adult phenology, demography, mobility and distribution in two syntopic ecotypes of Maculinea alcon (cruciata vs. pneumonanthe) (Lepidoptera: Lycaenidae) from Transilvania (Romania). Entomol. Romanica..

[82] Turlure C, Legrand D, Schtickzelle N, Baguette M (2016). Male disguised females: costs and benefits of female-limited dimorphism in a butterfly. Ecol. Entomol..

[83] Beldade P, Brakefield PM (2002). The genetics and evo-devo of butterfly wing patterns. Nat. Rev. Genet..

[84] Sielezniew M, Deoniziak K, Dziekańska I, Nowicki P (2019). Dispersal in a metapopulation of the critically endangered Danube Clouded Yellow butterfly *Colias myrmidone*: implications for conservation. J. Insect Conserv..

[85] Benedick S (2007). Butterfly dispersal and longevity in unlogged and selectively logged forest. Sepilok Bull..

[86] Molleman F, Zwaan BJ, Brakefield PM, Carey JR (2007). Extraordinary long life spans in fruit-feeding butterflies can provide window on evolution of life span and aging. Exp. Gerontol..

