# Supplementary material for: Sexual differences in age-dependent survival and life span of adults in a natural butterfly population
Source: Sci Rep. 2020 Jun 25;10:10394. doi: 10.1038/s41598-020-66922-w (PMC7316833; doi:10.1038/s41598-020-66922-w)
Supplement: Supplementary file 1 — Supplementary Information. [file 41598_2020_66922_MOESM1_ESM.pdf]

Supplementary information

Sexual differences in age-dependent survival and life span of adults in a natural butterfly population

Marcin Sielezniew[1*], Agata Kostro-Ambroziak[1], Ádám Kőrösi[2,3]

[1] Laboratory of Insect Evolutionary Biology and Ecology, Faculty of Biology, University of Bialystok, Ciołkowskiego 1J, 15-245 Białystok, Poland

[2] MTA-ELTE-MTM Ecology Research Group, Pázmány Péter s. 1/C., Budapest 1117, Hungary

[3] Theoretical Evolutionary Ecology Group, Department of Animal Ecology and Tropical Biology, Biocenter, University of Würzburg, Emil-Fischer str. 32, 97074 Würzburg, Germany

*corresponding author

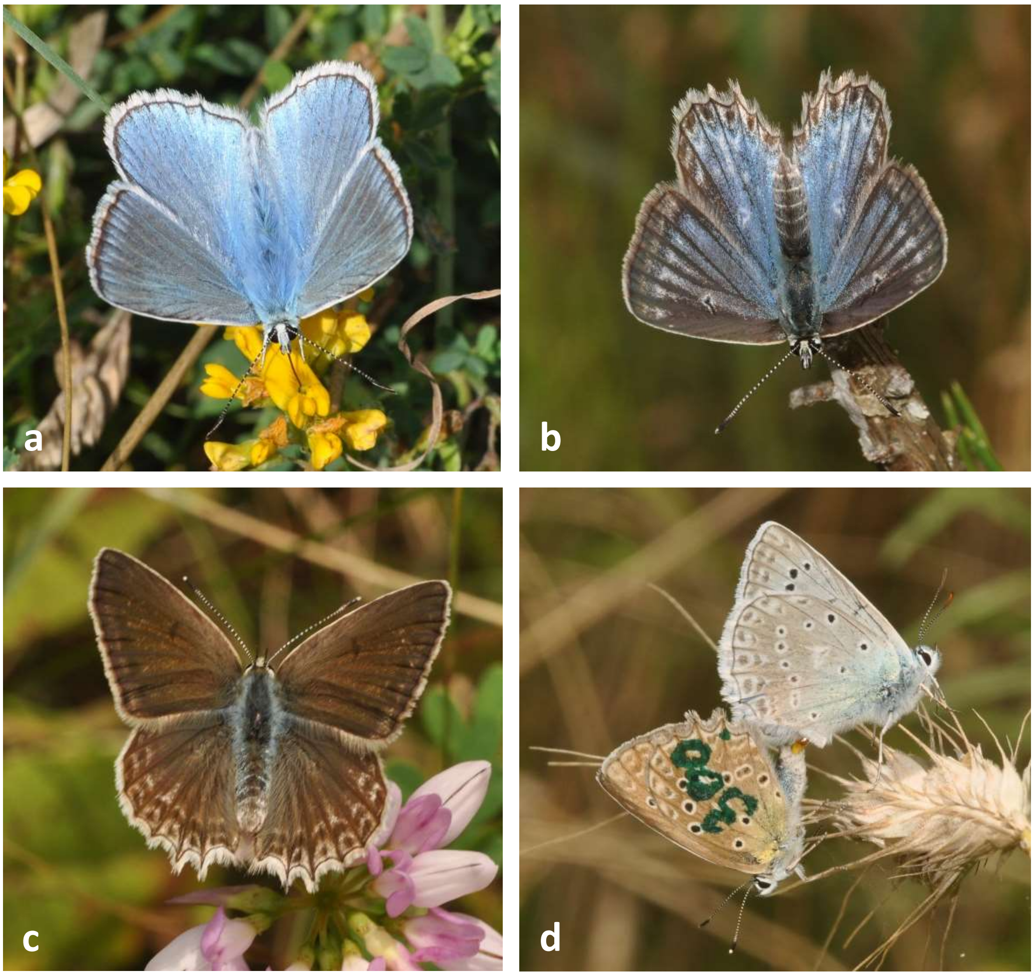

Figure S1. The study species *Polyommatus daphnis:* (a) a male; (b) a female of the blue form; (c) a female of the brown form; (d) a mating pair (with the female marked). Photo credits: Marcin Sielezniew.

Goodness of Fit (GOF) tests of the Cormack-Jolly-Seber models.

We carried out several GOF-tests using the MARK software. The so called 'RELEASE' GOF-test performs a series of χ^2-tests on the most general time-dependent model φ(~time)p(~time). It tests the assumptions of equal survival and equal catchability of marked individuals. The ratio of the overall χ^2 and the deviance degrees of freedom provides an estimate of the overdispersion parameter $\hat{c}$.

We also conducted a bootstrap GOF-test. We simulated 200 datasets using the parameter estimates of the model φ(~time)p(~time) for each sex, then we estimated the overdispersion parameter $\hat{c}$ by (i) dividing model deviance with the mean deviance of the simulated datasets and (ii) dividing model $\hat{c}$ with the mean $\hat{c}$ of the simulated datasets.

Thus we obtained three estimates of $\hat{c}$ for both males and females and we used the largest value of them in the following.

Nor the overall RELEASE GOF-test neither any of TEST 3 were significant. However, TEST 2 for the 13. sampling occasion in males (χ^2=19.71, d.f.=5, p=0.001) and for the 11. sampling occasion in females (χ^2=16.24, d.f.=4, p=0.003) were significant. This indicates some heterogeneity in catchability among individuals in these cohorts. TEST 2 in RELEASE is known to be a test of 'equal catchability' and the reason behind its violation can be temporary emigration, i.e. some individuals leave the sampling area and are not available for capture for a few sampling occasions and later return again. Since no females were captured on the first sampling day, the 11. sampling occasion for females is equivalent to the 12. occasion in males. These tests suggest that some females might have emigrated from the sampling area on/right before the 12. occasion and some males might have followed them on/right before the 13. occasion, but later most of these individuals returned. Interestingly, population size reached its peak around these two sampling occasions and sex ratio was 1:1 at this time of the sampling period. Without any observations of the butterflies outside of the study area, this remains a speculation though.

However, since the test showed significant violation of the model assumption for only one cohort from 33 (males) and 37 (females) cohorts, we continued using the CJS model.

In case of males, all estimates of $\hat{c}$ were below 1, which means no overdispersion (or extra-binomial noise). For females 2 values were below 1 (Table S1) and we used the largest value to adjust the models.

Table S1. Estimates of $\hat{c}$ for females and males data using different methods.

Method of estimating $\hat{c}$	females	males
X^2 / d.f.	1.07	0.997
Deviance / mean deviance	0.945	0.988
$\hat{c}$ / mean $\hat{c}$	0.998	0.93

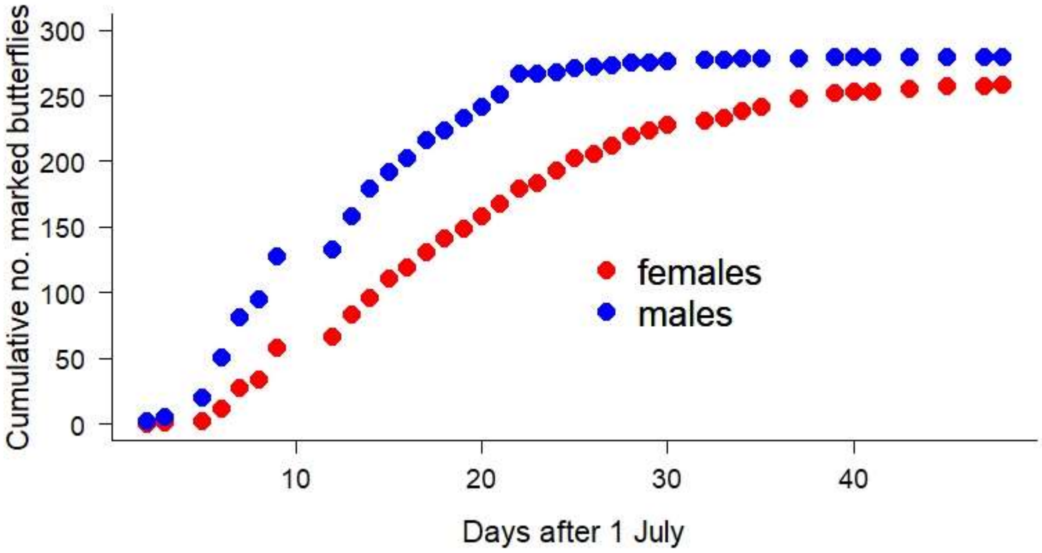

Figure S2. Cumulative number of marked butterflies during the sampling period.

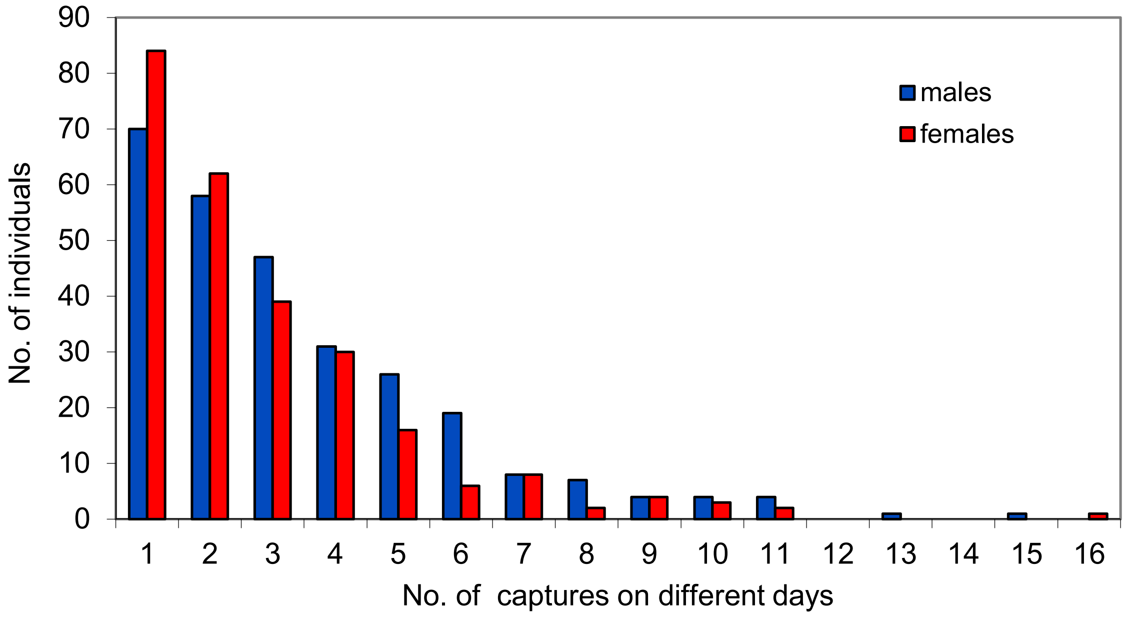

Figure S3. Frequency distribution of number of sampling occasions (only on different days) on which individuals were captured.

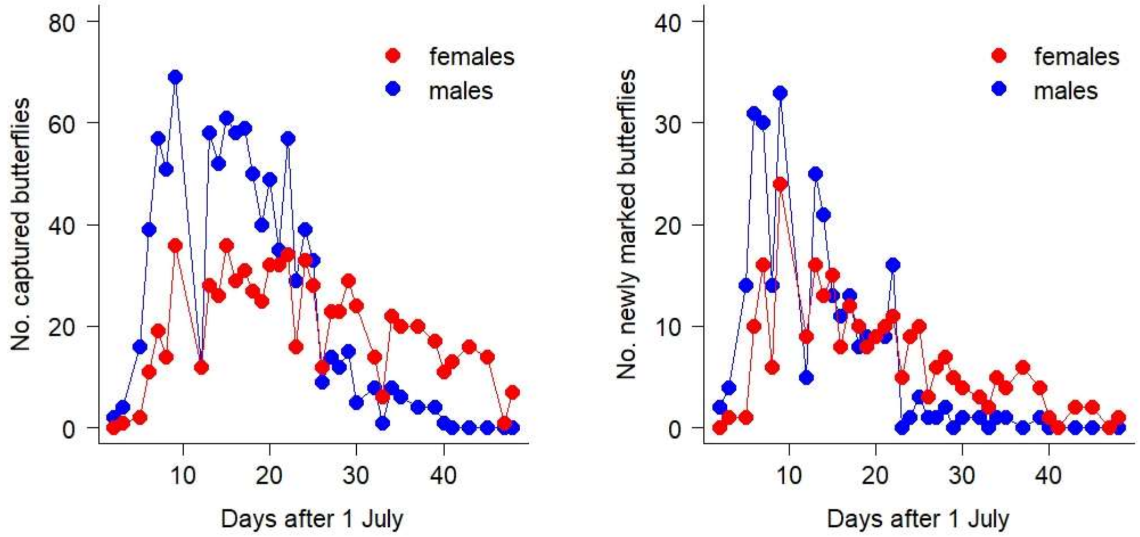

Figure S4. The number of captured and marked butterflies (males and females) on each day of the sampling season.
